# Right and left ventricular blood pool T2 ratio on cardiac magnetic resonance imaging correlates with hemodynamics in patients with pulmonary hypertension

**DOI:** 10.1186/s13244-023-01406-9

**Published:** 2023-04-15

**Authors:** Mei Deng, Anqi Liu, Wenqing Xu, Haoyu Yang, Qian Gao, Ling Zhang, Yanan Zhen, Xiaopeng Liu, Wanmu Xie, Min Liu

**Affiliations:** 1 Chinese Academy of Medical Sciences and Peking Union Medical College, Beijing, 100005 China; 2 Department of Radiology, Peking University China-Japan Friendship School of Clinical Medicine, Beijing, 100191 China; 3 Department of Pulmonary and Critical Care Medicine, China-Japan Friendship Hospital, Beijing, 100029 China; 4 Department of Radiology, China-Japan Friendship Hospital, No. 2 Yinghua Dong Street, Hepingli, Chao Yang District, Beijing, 100029 China; 5 Department of Cardiovascular Surgery, China-Japan Friendship Hospital, Beijing, 100029 China

**Keywords:** Pulmonary hypertension, Cardiac magnetic resonance imaging, T1 Mapping, T2 Mapping, Hemodynamics, Oxygen saturation

## Abstract

**Objectives:**

Our objective is to compare the right/left ventricular blood pool T1 ratio (RVT1/LVT1), and right/left ventricular blood pool T2 ratio (RVT2/LVT2) on Cardiac Magnetic Resonance Imaging (CMR) between patients with pulmonary hypertension (PH) and normal controls, to analyze the correlation of RVT1/LVT1, RVT2/LVT2 and hemodynamics measured with right heart catheterization (RHC) in patients with PH.

**Methods:**

Forty two patients with PH and 40 gender-and age-matched healthy controls were prospectively included. All patients underwent RHC and CMR within 24 h. The right and left ventricular blood pool T1 and T2 values were respectively measured, and RVT1/LVT1 and RVT2/LVT2 between the PH group and the healthy control were compared. Meanwhile, the correlation between RVT1/LVT1, RV/LVT2 ratio and hemodynamic parameters in patients with PH respectively was analyzed.

**Results:**

In the control group, RVT2 was significantly lower than LVT2 (*t* = 6.782, *p* < 0.001) while RVT1 also was lower than LVT1 (*t* = 8.961, *p* < 0.001). In patients with PH, RVT2 was significantly lower than LVT2 (*t* = 9.802, *p* < 0.001) while RVT1 was similar to LVT1 (*t* =  − 1.378, *p* = 0.176). RVT2/LVT2 in the PH group was significantly lower than that in the control group (*p* < 0.001). RVT1/LVT1 in PH patients increased in comparison with the control group (*p* < 0.001). RVT2/LVT2 negatively correlated with pulmonary vascular resistance (*r* =  − 0.506) and positively correlated with cardiac index (*r* = 0.521), blood oxygen saturation in Superior vena cava, right atrium, right ventricle and pulmonary artery (*r* = 0.564, 0.603, 0.648, 0.582).

**Conclusions:**

RVT2/LVT2 on T2 mapping could be an additional CMR imaging marker that may assist to evaluate the severity of PH.

## Key points

- Ventricular blood pool T1 and T2 values on T1 and T2 Mapping on four-chamber slice have good repeatability.
- LVT2 is higher than RVT2 in both normal control and PH patients. However, RVT2 in PH patients has a significant reduction, compared with the normal control.
- RVT2/LVT2 ratio in PH patients correlated with hemodynamics such as PVR, CI, blood oxygen saturation in SVC, right atrium, right ventricle and pulmonary artery

## Introduction

Pulmonary hypertension (PH) is a complex chronic disorder of the pulmonary circulation that defined as an increase in mean pulmonary arterial pressure (MPAP) > 20 mmHg and pulmonary vascular resistance (PVR) > 2Wu at rest as assessed by right heart catheterization (RHC). Currently, RHC is the "gold standard" for the diagnosis and evaluation of pulmonary hypertension [1]. As the free-radiation imaging technique, cardiac magnetic resonance imaging (CMR) is an accurate and reproducible method for the assessment of heart size, morphology, and function, and permits the noninvasive assessment of blood flow and it has been increasingly used to non-invasively monitor right ventricular function and pulmonary hemodynamics in PH [2–4].

T1 and T2 mapping is a quantitative technique for measuring spin–lattice and transverse relaxation time (T2) of magnetic resonance imaging (MRI). Both T1 and T2 values are the physiological tissue property that can be exploited with MRI to generate contrast between normal and diseased tissues. To date, Tissue characterization by native T1 mapping can serve as a non-invasive method for the assessment of myocardial fibrosis on CMR [5, 6]. García-Álvarez et al. [7] reported that CMR-measured native T1 and Eq-ECV at the RV insertion points increased in the early stages of chronic PH and correlated with hemodynamics and RV dysfunction in an experimental porcine model of chronic PH. In patients with chronic thromboembolic pulmonary hypertension (CTEPH), native septal T1 values show good correlations with right ventricular function and hemodynamics before BPA [8, 9]. T2 mapping is a useful and better method for the detection of diffuse myocardial edema than that of conventional T2WI [10–12]. Moreover, some studies have shown that blood T2 is sensitive to the level of blood oxygenation and quantitative T2 Mapping is a novel, non-invasive method to estimate blood O2 saturation [13–15]. Emrich et al. [16] reported that right and left ventricular blood pool T2 ratio can qualify left-to-right shunts in patients with known L-R shunt diseases. During clinical practice, we found a prominent intensity difference in the right ventricular (RV) and left ventricular (LV) blood pool T2 Map in patients with PH. However, this phenomenon and its clinical significance have not been clarified yet. Therefore, the purpose of our study was to compare LV and RV blood pool T1 and T2 values in patients with PH and healthy volunteers, to explore the correlation between RV to LV blood pool T1 and T2 ratio and hemodynamics in PH patients, and to analyze the significance of ventricular blood pool T1 and T2 Map in evaluating PH.

## Materials and methods

### Cohort and design

This study complied with the Declaration of Helsinki and was approved by the ethics committee of our hospital (IRB No. 2022-KY-048). Informed consent was obtained from all participants or their families. From January 2020 to January 2022, patients with chronic thromboembolic pulmonary hypertension (CTEPH) (20 males, mean age = 47.5 ± 9.1 years) and gender- and age-matched healthy volunteers as the control group (15 males, mean age = 47.1 ± 10.8 years old) were prospectively included. CTEPH was diagnosed by RHC with a radionuclide pulmonary ventilation-perfusion scan and computed tomography pulmonary angiography (CTPA). CMR and RHC were finished within 24 h. Patients who were older than 18 years and less than 70 years were included. Patients with congenital heart diseases or malignancies were excluded. Patients without complete hemodynamic data of RHC or patients whose mean pulmonary artery pressure (MPAP) < 20 mmHg were excluded. Patients who did not complete CMR or had poor imaging quality were also excluded.

### Cardiac magnetic resonance imaging

All participants underwent CMR within 24 h before RHC using a 1.5 Tesla clinical scanners (MAGNETOM Area, Siemens Healthcare, Germany) in the supine position with an 18-channel body coil without supplemental oxygen. CMR was acquired during end-expiratory breath holds with retrospective electrocardiographic gating. Using a balanced steady-state free precession sequence with ECG gating, a true steady-state precession fast imaging sequence (TrueFISP T2 Map) was used to scan short-axis slices and long-axis four-chamber slice with the scanning parameters: TE/TR = 193.27 ms/1.06 ms, Flip angle 70°, Slice thickness 8.0 mm, voxel = 1.9 × 1.9 × 8.0 mm3. Field of view = 360 × 256 mm, GRAPPA = 2, T2 preparation: 0 ms, 25 ms, 55 ms, cardiac delay time TD = 517 ms, phase partial Fourier 7/8. Each slice was 8-mm thick with a 2-mm gap.

T1-mapping was obtained with a modified look-locker inversion-recovery (MOLLI) sequence with a single-shot balanced steady-state free precession (bSSFP) readout including the short-axis slices and long-axis four-chamber slice as T2 Map using the following parameters: TE/TR = 280.56/1.14 ms, Flip angle = 35°, Voxel size = 1.4 × 1.4 × 8 mm^3^, Field of view = 360 × 256 mm, GRAPPA = 2, 24 reference lines, cardiac delay time TD = 435 ms, and phase partial Fourier 7/8. Each slice was 8-mm thick with a 2-mm gap. 2 inversions pulses with 5 and 3 images were acquired after each inversion pulse respectively and 3 heart beats to recover before the 2nd inversion If necessary, shimming and center frequency adjustments were performed to generate images free from off-resonance artifacts.

### Measurement of ventricular blood pool T1 and T2

**Fig. 1:**
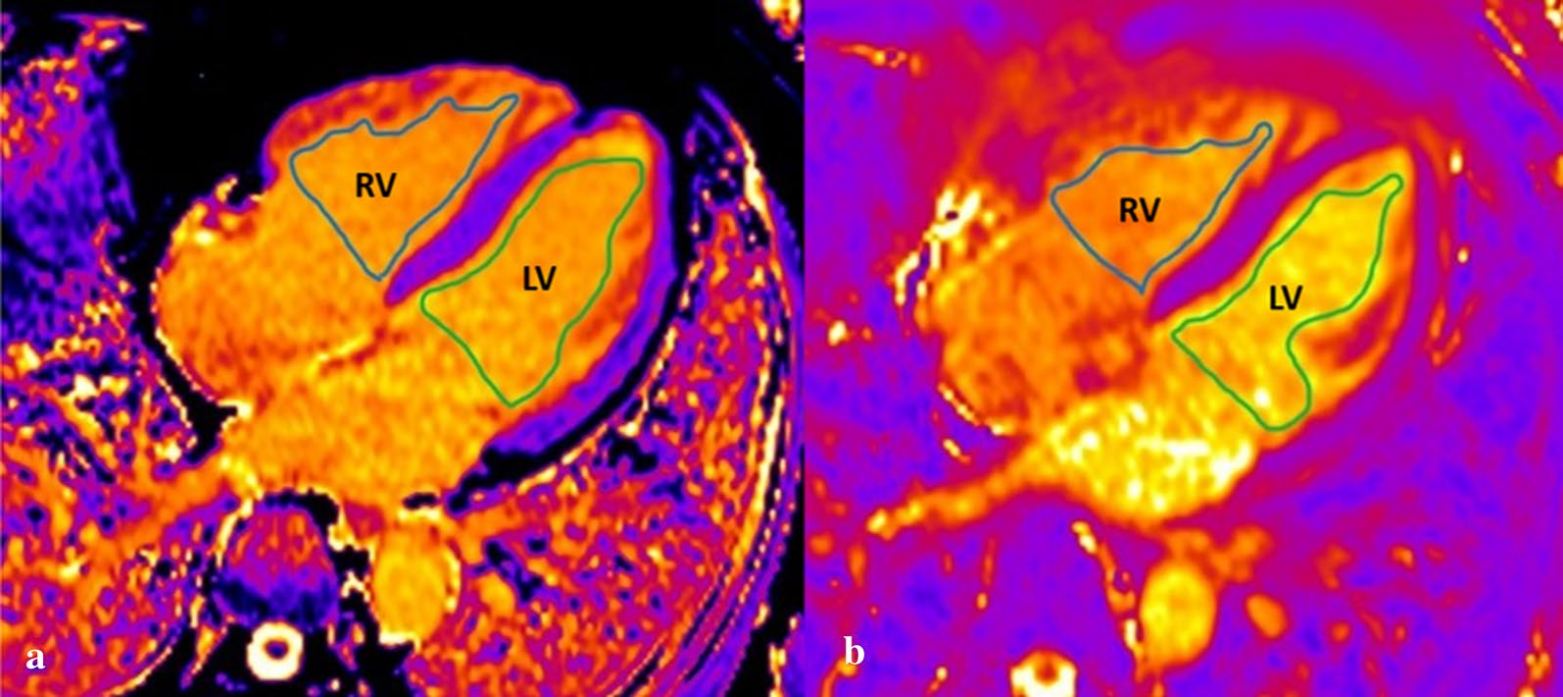
Measurement of RV and LV blood pool T1 (**a**) and T2 (**b**) the 4-chamber cardiac view of T1 map and T2 map

Right and left ventricular blood pool T1 and T2 values were respectively measured on the four-chamber slice of T1 map and T2 map with an Argus (SyngoVia workstation, Siemens Healthcare Sector, Forchheim, Germany). Region of interest (ROI) of the left and right ventricular blood pool were delineated along the endomyocardium on the four-chamber T1 Map and T2 Map (Fig. 1). To eliminate bias, right and left ventricular blood pool T1 and T2 values were analyzed independently by two observers who were blinded to other clinical information, and then one observer analyzed right and left ventricular blood pool T1 and T2 a second time after 4 weeks. The Right and left ventricular blood pool T1 ratio (RVT1/LVT1 ratio) and T2 ratio (RV/LV T2 ratio) were calculated from the mean value of three measurements.

### Right-heart catheterization

All PH patients underwent RHC. A Swan-Ganz standard thermodilution pulmonary artery catheter was placed at the right inferior pulmonary artery. The hemodynamics included pulmonary arterial pressure (PAP), pulmonary arterial wedge pressure (PAWP) and pulmonary vascular resistance (PVR). Cardiac Output (CO) and Cardiac Index (CI) were determined using the Fick method.

### Statistical analysis

Statistical analysis was performed using SPSS v26.0 (SPSSInc, Chicago, IL, USA). Normally distributed measurement data were described as mean ± standard deviation (SD). Non-normally distributed data were described by the median and interquartile range (IQR). Bland–Altman plot and intraclass correlation coefficient were used to assess the reproducibility of right and left ventricular blood pool T1 (RVT1, LVT1) and T2 (RV2, LVT2). The RV/LV T2 ratio was compared between the CTPEH and the control groups by t or U tests. Spearman's rank correlation analysis was performed between RV/LV T2 ratio and hemodynamic data in CTEPH patients. *p* < 0.05 means the difference is statistically significant.

## Results

### Baseline characteristics

Eighty two participants (35 males, mean age = 47.3 ± 9.9 years), including 42 patients in the PH group (20 males, mean age = 47.5 ± 9.1 years) and 40 healthy volunteers in the control group (15 males, mean age = 47.1 ± 10.8 years old) enrolled this study. Table 1 shows the basic clinical information of all participants. Gender and age were comparable between the PH group and the control group.

**Table 1:** Clinical information of patients with PH and the normal control

	PH group	Control group	*x2/t* (*p*)
Cases (n)	42	40	
Gender (male/female)	20/22	15/25	0.858 (*p* = 0.354)
Age (years)	47.5 ± 9.1	47.1 ± 10.8	− 0.171 (*p* = 0.865)
Height (cm)	1.65 ± 0.08	1.68 ± 0.07	1.930 (*p *= 0.062)
Weight (kg)	66.6 ± 10.4	72.9 ± 12.6	2.656 (*p* = 0.010)
BMI (kg/m^2^)	24.5 ± 3.0	25.8 ± 4.4	1.623 (*p* = 0.108)
Heart rate (bpm)	70.9 ± 13.3	74.1 ± 12.5	1.147 (*p* = 0.255)
Systolic BP (mmHg)	116.2 ± 16.1	111.0 ± 8.7	− 1.804 (*p* = 0.075)
Diastolic BP (mmHg)	77.4 ± 13.9	75.7 ± 9.5	− 0.664 (*p* = 0.509)
Diagnosis	CTEPH		
Treatment	PEA		
*Pulmonary hemodynamic parameters*			
Systolic PAP (mmHg)	78.7 ± 19.9		
Diastolic PAP (mmHg)	26.8 ± 9.3		
Mean PAP (mmHg)	43.7 ± 11.6		
PVR (Wood)	11.9 ± 6.1		
PAWP (mmHg)	10.0 ± 2.9		
CI (L/min/m^2^)	1.9 ± 0.7		
CO(L/min)	3.3 ± 1.1		
SVC SO_2_ (%)	28.9 ± 8.9		
RA SO_2_ (%)	65.8 ± 10.1		
RV SO_2_ (%)	65.0 ± 10.8		
PA SO_2_ (%)	65.1 ± 11.3		
FA SO_2_ (%)	98.1 ± 2.7		

*PH* Pulmonary hypertension, *BMI* Body Mass Index, *BP* Blood pressure, *CTEPH* Chronic thromboembolic pulmonary hypertension, *PEA* Pulmonary thromboendarterectomy, *PAP* Pulmonary artery pressure, *PVR* Pulmonary vascular resistance, PAWP Pulmonary arterial wedge pressure, *CI* Cardiac Index, *CO* Cardiac Output, *SO*_*2*_ Oxygen saturation, *SVC* Superior vena cava, *RA* Right atrium, *RV* Right ventricle, *PA* Pulmonary artery, *FA* Femoral artery

### Repeatability of ventricular blood pool T1 and T2 measurements

**Fig. 2:**
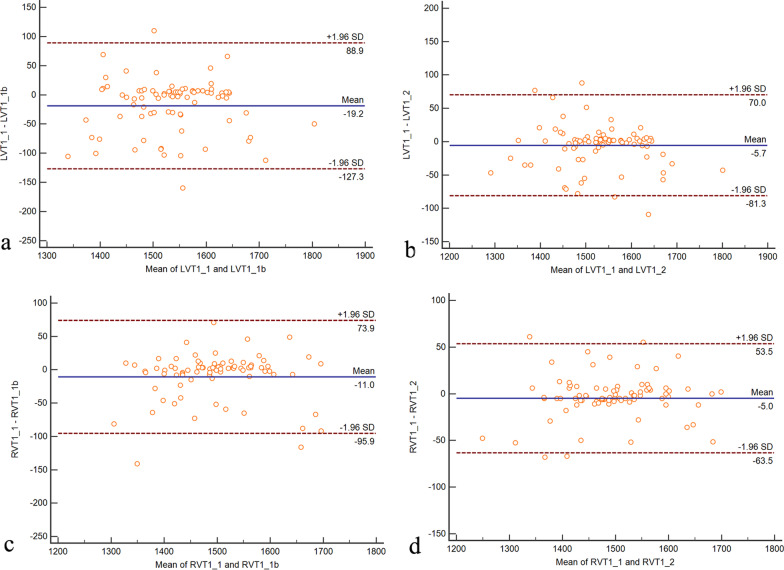
A Bland–Altman plot of left ventricular blood pool T1 (LVT1) and right ventricular blood pool T1(RVT1)measurements. **a** The intra-observer mean difference for LVT1 was  − 19.2 (95% confidence interval [CI] − 127.3 to 88.9). **b** The inter-observer mean difference for LVT1 was − 5.7 (95% CI − 81.3 to 70.0). **c** The intra-observer mean difference for RVT1 was − 11.0 (95% CI − 72.9 to 95.9). **d** The inter-observer mean difference for LVT1 was − 5.0 (95% CI − 63.6 to 53.5)

A Bland–Altman plot of LVT1 is shown in Fig. 2. The mean difference between the first and second readings by one reader was -19.2, while the mean + 1.96 SD was 88.9 and the mean − 1.96 SD was − 127.3. The mean difference between the two readers was − 5.7, while the mean ± 1.96SD was 70 and the mean-1.96SD was − 81.3. A Bland–Altman plot of RVT1 is shown in Fig. 2. The mean difference between the first and second readings by one reader was − 11.0, while the mean + 1.96SD was 73.9 and the mean-1.96SD was − 95.9. The mean difference between the two readers was − 5.0, while the mean + 1.96SD was 53.5 and the mean-1.96SD was − 63.6. Intra-class correlation coefficient (ICC) for LVT1 and RVT1 respectively was 0.954 and 0.972.

**Fig. 3:**
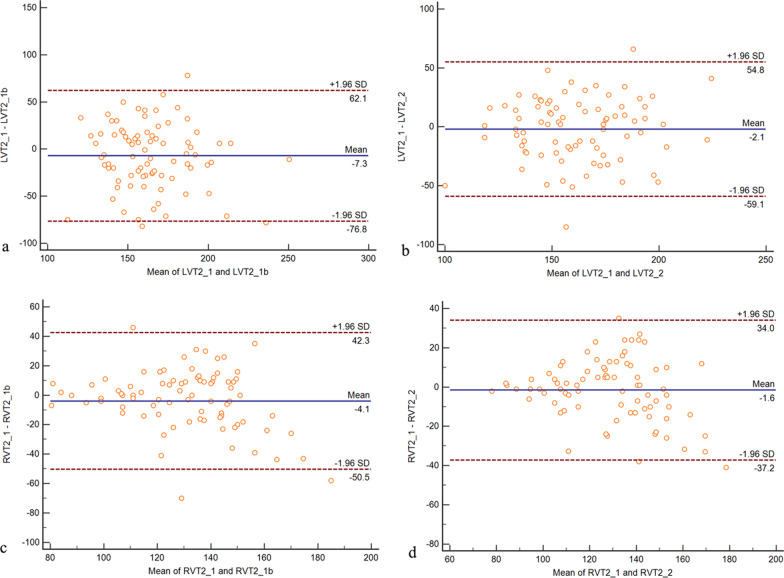
A Bland–Altman plot of left ventricular blood pool T2 (LVT2) and right ventricular blood pool T2 (RVT2)measurements. **a** The intra-observer mean difference for LVT2 was  − 7.3 (95% CI − 76.8 to 62.1). **b** The inter-observer mean difference for LVT2 was − 2.1 (95% CI − 51.9 to 54.8). **c** The intra-observer mean difference for RVT2 was − 4.1 (95% CI − 50.5 to 42.3). **d** The inter-observer mean difference for LVT2 was − 1.6 (95% CI − 37.2 to 34.0)

A Bland–Altman plot of LVT2 is shown in Fig. 3. The mean difference between the first and second readings by one reader was − 7.3, while the mean + 1.96SD was 62.1 and the mean-1.96SD was − 76.8. The mean difference between the two readers was − 2.1, while the mean ± 1.96SD was 54.8 and the mean-1.96SD was − 51.9. A Bland–Altman plot of RVT2 is shown in Fig. 3. The mean difference between the first and second readings by one reader was − 4.1, while the mean + 1.96SD was 42.3 and the mean-1.96SD was − 50.5. The mean difference between the two readers was − 1.6, while the mean + 1.96SD was 34.0 and the mean-1.96SD was − 37.2. Intra-class correlation coefficient (ICC) for LVT2 and RVT2 respectively was 0.934 and 0.953.

### Comparison of ventricular blood pool T1 and T2 values

**Fig. 4:**
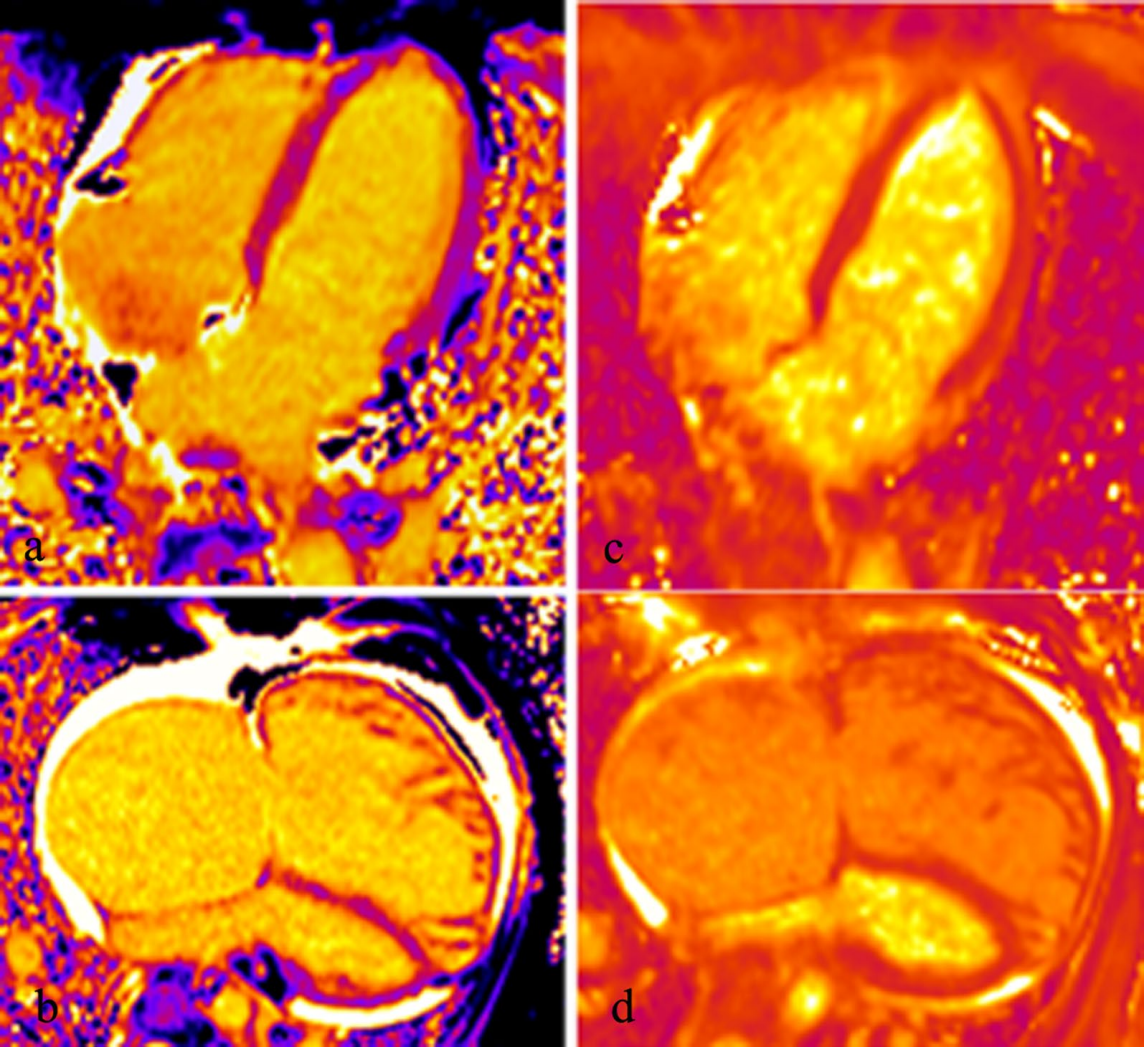
The representative T1 and T2 Maps in a healthy volunteer and a patient with PH. **a** the long-axial four-chamber T1 Map in a 42-year male healthy volunteer. **b** The long-axial four-chamber T1 Map in a 42-year male patient with PH. **c** the long-axial four-chamber T2 Map in a 42-year male healthy volunteer. **d** the long-axial four-chamber T2 Map in a 42-year male patient with PH

**Fig. 5:**
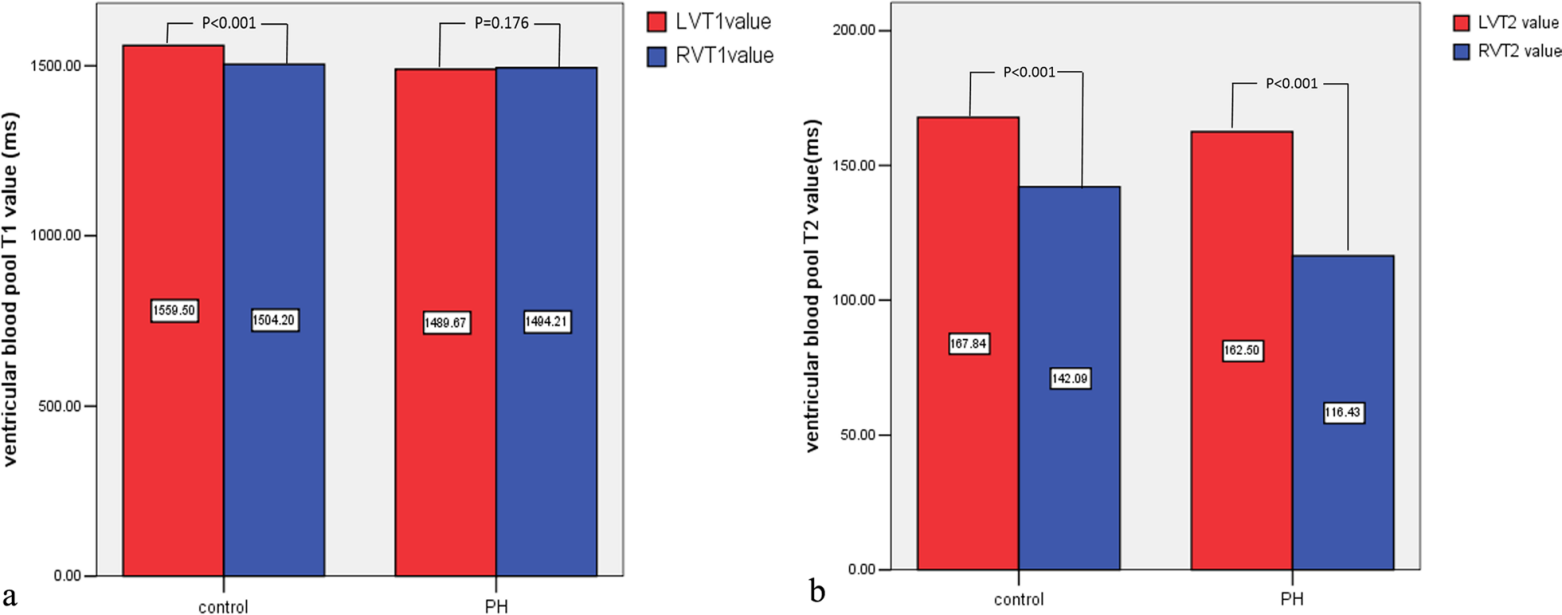
Comparison of ventricular blood pool T1 and T2 in healthy volunteers and PH patients. **a** LVT1 is higher than RVT1 in healthy volunteers and LVT1 is comparable to RVT1 in PH patients. **b** LVT2 is higher than RVT2 in healthy volunteers and PH patients

Figure 4 shows the representative T1 and T2 Maps in a patient with CTEPH and a normal control. As shown in Fig. 5, RVT2 was significantly lower than LVT2 (*t* = 9.802, *p* < 0.001) while RVT1 was similar to LVT1 in patients with PH (*t* =  − 1.378, *p* = 0.176). In normal control group, RVT2 was significantly lower than LVT2 (*t* = 6.782, *p* < 0.001) while RVT1 also was lower than LVT1 (*t* = 8.961, *p* < 0.001).

**Fig. 6:**
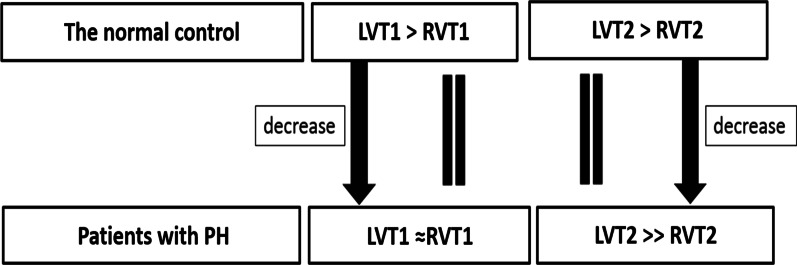
The alteration of ventricular blood pool T1 and T2

Table 2 shows that RVT1 in the PH group was comparable to RVT1 in the control group (*p* = 0.591) while LVT1 in the PH group was lower than LVT1 in the control group (*p* < 0.001). RVT1/LVT1ratio in PH patients increased in comparison with the control group (*p* < 0.001). RVT2 in PH patients significantly decreased in comparison with RVT2 in the control (*p* < 0.001), however, there was no significant difference in LVT2 values between the two groups (*p* = 0.328). The RVT2/LVT2 ratio in the PH group were significantly lower than that in the control group (*p* < 0.001). The alteration of ventricular blood pool T1 and T2 is shown in Fig. 6.

**Table 2:** Ventricular Blood pool T1 and T2 in patients with PH and the normal control

Ventricular blood pool T1/T2 mapping	PH group (*n* = 42)	Control group (*n* = 40)	*t* (*p*)
RV T1 (ms)	1494.21 ± 102.50	1504.20 ± 58.05	0.539 (0.591)
LV T1 (ms)	1489.67 ± 107.32	1559.50 ± 56.93	3.654 (< 0.001)*
RVT1/LVT1 ratio	0.99 ± 0.04	0.96 ± 0.03	4.018 (< 0.001)*
RV T2 (ms)	116.43 ± 20.85	142.09 ± 15.13	6.263 (< 0.001)*
LV T2 (ms)	162.50 ± 25.01	167.84 ± 23.15	0.985 (0.328)
RVT2/LVT2 ratio	0.74 ± 0.16	0.86 ± 0.13	3.547 (= 0.001)*

*PH* Pulmonary hypertension, *RV* Right ventricular, *LV* Left ventricular

*p <  = 0.001

### RV/LV T1 and T2 ratio and hemodynamics in patients with PH

As shown in Table 3, RVT2/LVT2 ratio negatively correlated with SPAP (*r* = − 0.310), DPAP (*r* =  − 0.372), MPAP (*r* =  − 0.343), PVR (*r* =  − 0.506,). It was positively correlated with CO (*r* = 0.489), CI (*r* = 0.521), SVC oxygen saturation (*r* = 0.564), right atrial oxygen saturation (*r* = 0.603), right ventricular oxygen saturation (*r* = 0.648), and pulmonary artery oxygen saturation (*r* = 0.582). RVT1/LVT1ratio only had a weak negative correlation with PAWP (*r* =  − 0.311).

**Table 3:** Correlation of ventricular blood pool T1/T2 ratio with hemodynamics in PH patients

Hemodynamics	RVT1/LVT1 ratio *R* (*p*)	RVT2/LVT2 ratio *R* (*p*)
Systolic PAP (mmHg)	0.078 (0.718)	− 0.310 (0.046)
Diastolic PAP (mmHg)	0.103 (0.515)	− 0.372 (0.015)
Mean PAP (mmHg)	0.090 (0.572)	− 0.343 (0.026)
PVR (Wood)	0.130 (0.411)	− 0.506 (0.001)
CO(L/min)	0.052 (0.742)	0.489 (0.001)
CI (L/min/m^2^)	0.038 (0.813)	0.521 (< 0.001) *
PAWP (mmHg)	− 0.311 (0.045)	− 0.248 (0.114)
SVC SO_2_ (%)	0.051 (0.783)	0.564 (0.001)
RA SO_2_ (%)	0.040 (0.802)	0.603 (< 0.001) *
RV SO_2_ (%)	− 0.027 (0.866)	0.648 (< 0.001) *
PA SO_2_ (%)	− 0.045 (0.775)	0.582 (< 0.001) *
FA SO_2_ (%)	− 0.131 (0.408)	0.195 (0.216)

*PH* Pulmonary hypertension, *PAP* Pulmonary artery pressure, *PVR* Pulmonary vascular resistance, *PAWP* Pulmonary arterial wedge pressure, *CI* Cardiac Index, *CO* Cardiac Output, *SO*_*2*_ Oxygen saturation, *SVC* Superior vena cava, *RA* Right atrium, *RV* Right ventricle, *PA* Pulmonary artery, *FA* Femoral artery

*p < 0.001

## Discussion

There are several major findings in the present study. (I) Ventricular blood pool T1 and T2 measurements with T1 and T2 Mapping on CMR four-chamber view have good repeatability. (II). LVT2 is higher than RVT2 in both normal control and PH patients. However, RVT2 in PH patients has a significant reduction, compared with the normal control. (III) RVT2/LVT2 ratio in PH patients correlated with hemodynamics such as PVR, SVC, right heart and pulmonary artery oxygen saturation. (IV). In normal control, LVT1 is more than RVT1. Compared with the normal control, LVT1 decreased in patients with PH, however, RVT1/LVT1 ratio only has a weak correlation with PAWP.

Although myocardial T1 and T2 mapping have been proven excellent repeatability and is widely used to assess myocardial injury under various pathological conditions, we first studied T1 and T2 value of ventricular blood pool in patients with CTEPH. To accurately measure RV and LV blood pool T1 and T2, outlining the ventricular blood pool must avoid the muscle trabecula and papillary muscle. In the current study, blood pool T1 and T2 demonstrate better repeatability, which is the foundation of further research.

We first described the characteristics of RV and LV blood pool T2 Map in PH patients and healthy volunteers. Theoretically, relaxation times of blood are therefore a function of both the quantity of hemoglobin and the oxygen saturation [sO_2_] [17, 18]. The magnetic susceptibility of hemoglobin depends on its oxygenation state. Oxyhemoglobin has a low spin and a high signal on T2, while deoxyhemoglobin has a high spin and a low signal on T2. The higher the blood oxygen saturation is, the higher the intracellular hemoglobin content is, and the blood T2 will increase. Thus, T2 is sensitive to blood oxygenation and the alteration of blood oxygen saturation will affect the blood T2 value [14, 19–21]. Previous studies quantified the relationship between blood T2 value and blood oxygen saturation by the Luz-Meiboom chemical exchange model, which showed a positive correlation between blood T2 value and blood oxygen saturation. The blood oxygen saturation value was calculated by measuring the T2 value in vitro and in animal experiments [14]. In healthy people, arterial oxygen saturation (SaO2) in the left heart is higher than venous oxygen saturation (SvO_2_) in the right heart, which can explain why LVT2 is more than RVT2 in normal condition.

In PH patients, pulmonary vascular remodeling leads to a progressive increase in pulmonary vascular resistance and a worsening right heart function, resulting in a further reduced blood oxygen saturation and evaluated deoxyhemoglobin in the erythrocyte of SVC, the right atrium and right ventricle, pulmonary artery. Thus, RVT2 in PH patients significantly decreased compared with healthy control. However, in patients with precapillary PH, blood oxygen saturation and intracellular hemoglobin content in the erythrocyte of the left atrium and ventricle is less affected. Our patients with CTEPH belong to precapillary PH. That explains why LVT2 in normal control is comparable to LVT2 in CTEPH patients.

Emrich et al. [16] reported that RVT2/LVT2 ratio in L-R shunt patients (0.89 ± 0.07) in patients with the right ventricular disease (0.72 ± 0.10) was significantly higher than healthy volunteers (0.71 ± 0.09). In L-R shunt patients, RV blood oxygen saturation increased, leading to the increase in RVT2, but they did not evaluate the correlation of RVT2/LVT2 and blood oxygen saturation. RVT2/LVT2 ratio in our normal control seems to be higher than that in healthy volunteers reported by Emrich et al. [16]. We speculated this difference could be related to measurements on the different slices (the long-axis four-chamber slice in the current study and the short-axis slices in Emrich’s study). Importantly, our study indicated that RV/LV T2 ratio in CTEPH patients (0.74 ± 0.16) was significantly lower than that in normal control (0.86 ± 0.13). Furthermore, RV/LV T2 ratio in PH patients negatively correlated with PVR and positively correlated with right cardiac index, blood oxygen saturation in superior vena cava, right heart and pulmonary artery, which suggested ventricular blood pool T2 mapping noninvasive supply hemodynamics and could do help to differentiate PH secondary to systemic-to-pulmonary shunt and other types of PH.

T1 map showed that LVT1 was more than RVT1 in the normal control. Unexpectedly, compared with the normal control, LVT1 in patients with PH has a significant decrease while RVT1 between the PH group and normal control is comparable, and RVT1/LVT1 only has a weak correlation with PAWP. This suggested hemodynamics may have less effect on the ventricular blood pool T1. The possible mechanism which affects LVT1 but not RVT1 remains unclear.

### Study limitations

First, this was a single-center study performed at a large tertiary hospital, so the inherent limitations of this study design cannot be avoided. Second, our study only included patients with CTEPH, so further studies are needed to determine the relationship between ventricular blood pool T2 and hemodynamics, cardiac function and the prognosis in patients with other etiologies of PH. A non-contrast CMR protocol was applied, therefore, post-contrast T1 mapping could not be assessed. Since we cannot obtain the hemodynamics of healthy people with RHC, the cutoff value of blood pool RVT2/LVT2 for predicting PVR and MPAP remains unknown. Finally, all included patients with CTPEH underwent PEA, the correlation of the ventricular blood pool T2 value with the progress and prognosis of CTEPH patients remains for further research.

## Conclusions

Ventricular blood pool RVT2/LVT2 ratio on T2 mapping could be an additional new CMR imaging biomarker in addition to conventional CMR functional metrics that may assist to evaluate the severity of PH.

## Data Availability

The datasets generated during and/or analyzed during the current study are available from the corresponding author on reasonable request.

## Abbreviations

BPA: Balloon pulmonary angioplasty
CI: Cardiac index
CMR: Cardiac magnetic resonance imaging
CO: Cardiac output
CTEPH: Chronic thromboembolic pulmonary hypertension
CTPA: Computed tomography pulmonary angiography
ECV: Extracellular volume
LV: Left ventricle
MPAP: Mean pulmonary arterial pressure
MRI: Magnetic resonance imaging
PAWP: Pulmonary arterial wedge pressure
PEA: Pulmonary endarterectomy
PH: Pulmonary hypertension
PVR: Pulmonary vascular resistance
RHC: Right heart catheterization
RV: Right ventricle
SaO_2_: Arterial oxygen saturation
SvO_2_: Venous oxygen saturation
SVC: Superior vena cava

## Declarations

### Statistics and biometry

No complex statistical methods were necessary for this paper.
